# NF-κB/Rel-Mediated Regulation of the Neural Fate in *Drosophila*

**DOI:** 10.1371/journal.pone.0001178

**Published:** 2007-11-14

**Authors:** Savita Ayyar, Daniela Pistillo, Manuel Calleja, Anna Brookfield, Kelly Gittins, Claire Goldstone, Pat Simpson

**Affiliations:** 1 Department of Zoology, University of Cambridge, Cambridge, United Kingdom; 2 Centro de Biologia Molecular, Universidad Autonoma, Madrid, Spain; Baylor College of Medicine, United States of America

## Abstract

Two distinct roles are described for Dorsal, Dif and Relish, the three NF-κB/Rel proteins of *Drosophila,* in the development of the peripheral nervous system. First, these factors regulate transcription of *scute* during the singling out of sensory organ precursors from clusters of cells expressing the proneural genes *achaete* and *scute*. This effect is possibly mediated through binding sites for NF-κB/Rel proteins in a regulatory module of the *scute* gene required for maintenance of *scute* expression in precursors as well as repression in cells surrounding precursors. Second, genetic evidence suggests that the receptor Toll-8, Relish, Dif and Dorsal, and the caspase Dredd pathway are active over the entire imaginal disc epithelium, but *Toll-8* expression is excluded from sensory organ precursors. Relish promotes rapid turnover of transcripts of the target genes *scute* and *asense* through an indirect, post-transcriptional mechanism. We propose that this buffering of gene expression levels serves to keep the neuro-epithelium constantly poised for neurogenesis.

## Introduction

The proneural genes of the *achaete-scute (ac-sc)* and *atonal* families encode related transcription factors of the basic helix-loop-helix family that function as heterodimers together with the co-factor Daughterless/E2A/HEB/E2-2 [1]. The structure and function of these proteins is highly conserved throughout the animal kingdom. They are expressed in the neuro-epithelium during development and confer neural potential to cells through activation of neuronal precursor genes that regulate differentiation of neurons.

Single neuronal precursors are generated in a spaced array within domains of proneural gene expression through Notch-mediated lateral inhibition. This is a conserved process involving an indirect auto-regulatory loop whereby the proneural genes repress their own transcription. Cells chosen to be precursors sustain high threshold levels of expression and signal to the cells surrounding them causing them to gradually lose proneural gene expression [1]. Neuronal precursors move away from the neuro-epithelium and subsequently proneural genes are re-expressed. Successive waves of proneural gene expression thus allow the repeated generation of waves of neuronal precursors. This suggests that the neuro-epithelium is constantly poised to express the proneural genes in the absence of the inhibitory signal.

The array of sensory bristles on the *Drosophila* thorax is a useful paradigm for understanding the control of neuronal precursor development [2], [3]. In the imaginal disc *ac* and *sc* are expressed in small clusters of cells from which one or two sensory organ precursors (SOP) are singled out [4], [5], [6]. High levels of Ac-Sc in cells chosen to be SOPs activate neuronal-specific genes such as *asense (ase)* and *senseless (sens)* [7], [8], [9], [10], [11], [12], [13].

Culi and Modolell [2] described a regulatory element in the *sc* gene, the SOP element, which mediates lateral inhibition. It drives auto-regulation of *sc* in the SOP itself and is probably a target for repression in the inhibited cells. The enhancer bears binding sites for Ac-Sc/Da (E boxes) and a potential site for repression by the *E(spl)* bHLH proteins, targets of Notch signalling (N box). Three α boxes, motifs resembling the consensus binding sequence for transcription factors of the NF-κB/Rel family and a T-rich motif of unknown function were also found. Mutation of the α boxes in the *sc* SOP enhancer demonstrated a role for these sequences for maintenance of transcription in SOPs and for repression in inhibited cells [2]. This suggests a possible involvement of NF-κB/Rel proteins in SOP development. Three genes encoding NF-κB/Rel proteins are present in *Drosophila: dorsal (dl), Dorsal related immunity factor (Dif)* and *Relish (Rel)*. All three are involved in innate immunity and *dl* is also required for dorso-ventral polarity of the embryo [14], [15].

Toll-1 is known to initiate signalling leading to activation and nuclear translocation of the NF-κB/Rel proteins in *Drosophila* [15], [16]. Here we demonstrate a dual role for the three NF-κB/Rel proteins and one of the receptors of the Toll family, Toll-8, in the regulation of neurogenesis. We find that the NF-κB/Rel proteins promote the neural fate in SOPs. Activation of *sc* via the α boxes in the SOP enhancer might be direct. In addition, Toll-8 and Relish act to maintain low levels of expression over most of the epithelium of the target genes *sc, ase* and *sens,* by promoting rapid turnover of mRNA. This is mediated by a post-transcriptional mechanism affecting both RNA stability and translation. It appears to involve a heptamer nucleotide motif present in the coding regions similar to that described for the indirect regulation by NF-κB of *MyoD* and *Sox9* in mammalian cells [17]. We discuss the possibility that Relish regulates steady state levels of expression of genes required for the neuronal fate, so that the neuro-epithelium of the discs is constantly primed for neurogenesis.

## Results

### Mutants of *Toll-8* and *dorsal*, *Dif* and *Relish* display ectopic bristles at 18°C

**Figure 1:**
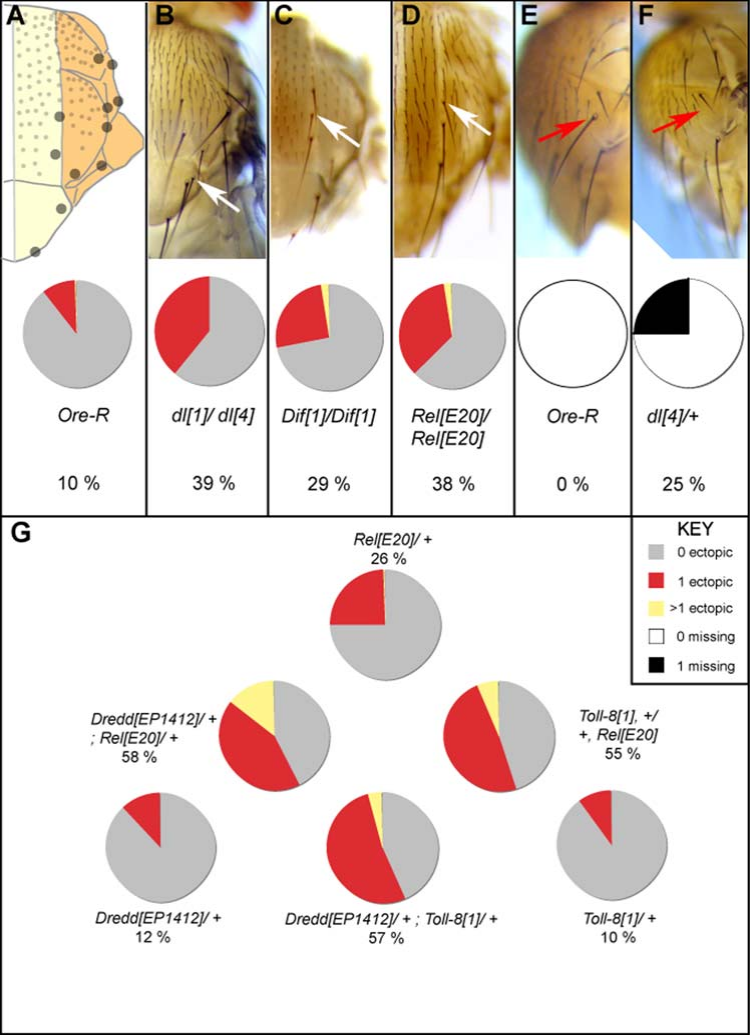
Loss-of-function mutants of *NFκB/Rel* genes display both loss and gain of macrochaetes on the notum. (A), a schematic representation of a wild-type heminotum, with the medial domain shaded yellow and the lateral domain orange. Large grey circles represent the positions of the eleven macrochaetes found on each heminotum. (B–D), heminota of flies homozygous mutant for *dorsal (dl^4^/dl^1^), Dif (Dif^1^/Dif^1^)* and *Relish (Rel^E20^/Rel^E20^);* white arrows indicate ectopic bristles. Below, pie charts represent the percentage of heminota displaying ectopic bristles from a total of 200. Red sectors indicate the percentage with one ectopic macrochaete, yellow sectors two or more ectopic macrochaetes (p>0.001 when compared with wild type). (E, F), heminota of *Ore-R* and *dl^4^/+* animals respectively, showing the lateral notum. Red arrows mark the pSA bristle, which is missing in a large fraction of *dl^4^/+* (and also *dl^4^/Df(2L)TW119, dl^−^ dif^−^* animals) (p>0.001). (G), the percentage of flies trans-heterozygous for various mutant combinations that display ectopic bristles (p>0.001).

The three NF-κB/Rel proteins Dorsal, Relish and Dif are detected ubiquitously in the imaginal epithelium [2], [18]. Since there are potential binding sites for these proteins in the *sc* promoter, we examined mutants of the three NF-κB/Rel genes for perturbations in the patterns of the large bristles (macrochaetes) of the notum. Flies homozygous for loss of function alleles of *dl, Dif* and *Rel* were found to display ectopic bristles on the notum in dorsocentral, scutellar and lateral regions (Figure 1B,C,D). The phenotype is in the form of one or more ectopic bristles per hemi-notum in a significant fraction of individuals for each genotype (typically 30% of heminota, n = 200 unless otherwise specified). It is observed in females reared at 18°C and is statistically significant (p<0.001). The phenotype is not seen at 25°C.

Animals heterozygous for these mutations also display bristle phenotypes at 18°C. While *Rel^E20^/+* and *Dif^1^/+* flies display ectopic dorsocentral bristles in the medial notum (26% and 8% respectively), bristles are missing in the lateral notum of *dl^4^/+* flies (Figure 1G and E–F). In addition, while 32% of heminota of double heterozygous *Dif^1^/+; +/Rel^E20^* flies display ectopic medial bristles, this phenotype is suppressed by heterozygosity for *dl,* so only 4% of heminota of animals triply heterozygous for *dl, Dif* and *Rel* display ectopic medial bristles. We also recovered imagos triply homozygous mutant for *dl, Rel* and *Dif*. Few animals of this genotype are viable, but the ten that did survive displayed a wild-type pattern of bristles. Taken together, the lack of observable phenotypes in the triple heterozygote and triple null suggests that the relative stoichiometric ratios between the three proteins are important in the final outcome on bristle patterning.

We also examined the activity of Toll-8, a member of the Toll-family of transmembrane receptors that initiate NF-κB/Rel signalling. A *Gal4* insertion in the *Toll-8* gene [19], [20], MD806, was identified in a screen for insertion lines reporting gene expression in the adult thorax [21]. *Toll-8* mutants were generated by imprecise excision of the transposon insertion in MD806. Here we employ *Toll-8^1^*, a null allele (Figure S1). *Toll-8^1^* mutants are viable and display a phenotype similar to *dl, Dif* and *Rel* mutants: 63% of heminota in homozygous females display ectopic macrochaetes at 18°C.

Previous work has shown that Dorsal and Dif are sequestered in the cytoplasm by the IκB factor Cactus whereas Relish has its own IκB domain [15], [22], [23], [24]. Two signalling pathways allowing nuclear translocation of these proteins are known to function in *Drosophila*. The first involves a phosphorylation cascade and the release of Dorsal and Dif from Cactus [15], [23]. The second pathway leads to proteolytic cleavage of Relish by the caspase Dredd to remove the IκB domain [25], [26]. Flies mutant for *Dredd* were found to display ectopic bristles at 18°C (not shown, 43% of heminota, n = 200). No involvement of *cactus* in bristle patterning could be detected from visible phenotypes in loss-of-function clones or in over-expression experiments (not shown).

Ectopic bristles were also observed in flies heterozygous for *Toll-8* (10% heminota) and this trait was used to detect interacting components. The bristle phenotype of flies simultaneously heterozygous for either *Dredd^EP1412^* and *Toll-8^1^* (57% heminota), or *Rel^E20^* and *Toll-8^1^* (55% heminota), was significantly enhanced compared to the single heterozygotes (*Dredd^EP1412^*/+ 12%, *Rel^E20^*/+ 26%; Figure 1G). No interaction was detected between *Toll-8^1^* and *Dif^1^* or between *Toll-8^1^* and *dl^4^* (not shown). These results suggest a link between Toll-8 and NF-κB/Rel signalling via the Dredd pathway.

We conclude from these observations that NF-κB/Rel proteins are active in the imaginal epithelium and that the receptor Toll-8 also plays a role. The mutant phenotypes suggest a function for Dorsal, Dif and Relish in repression of the neural fate.

### Over-expression experiments suggest that NF-κB/Rel proteins both promote and repress the neural fate

Ectopic bristles in the loss-of-function mutants are generally observed close to extant ones, suggesting that they arise from the proneural clusters of *ac-sc* expression. Therefore *sca-Gal4,* which is expressed in all proneural clusters on the notum, was used to over-express Rel, Dorsal and Dif.

**Figure 2:**
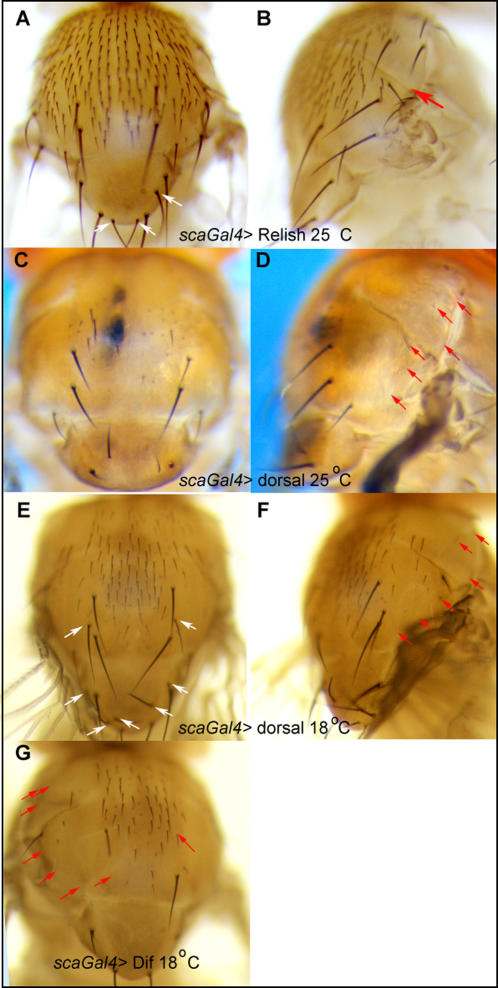
Over-expression of NF-κB/Rel proteins can result in both loss and gain of macrochaetes. *scabrous[537.4]Gal4* was used to over-express Relish (A, B) Dorsal (C–F) and Dif (G) in proneural clusters. This resulted in a loss of bristles in lateral regions (red arrows in B, D, F and G). Ectopic bristles were present in medial regions of animals after over-expression of Relish and Dorsal (white arrows in A, E).

Over-expression of full-length Rel at 25°C resulted in loss of bristles in the lateral notum and ectopic bristles in the medial notum (Figure 2A, B). Over-expression of Dorsal and Dif at 25°C resulted in a loss of bristles over much of the notum, although the loss is greater in the lateral notum (Figure 2C, 2G). However, over-expression of Dorsal at a lower temperature of 18°C also resulted in the generation of ectopic bristles on the medial notum in the dorsocentral and scutellar regions (Figure 2E). Note that the Gal4-UAS system used for these over-expression experiments is temperature-dependent: lower amounts of Gal4 and consequently less over-expressed Dorsal are synthesized at the lower temperature.

We conclude that the NF-κB/Rel proteins can promote or repress the neural fate in a region-specific manner and that differences are apparent between the activities of the three proteins.

### *Toll-8* expression is down-regulated in sensory organ precursors

**Figure 3:**
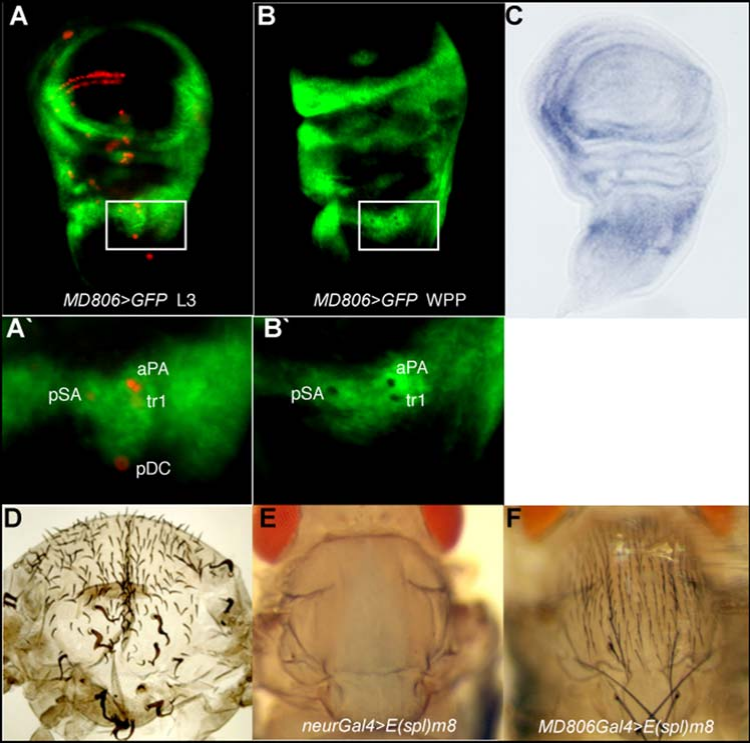
* Toll-8* is expressed at varying levels in the disc epithelium and expression is extinguished in the neural precursors. (A–B'), GFP expression driven by the *Toll-8[MD806]Gal4* driver in discs from third instar larvae (A, A') and white prepupae (B, B') respectively. Expression is strong over the lateral notum but is excluded from the sensory organ precursors, which are stained for *neuralized* activity (A101, anti-β galactosidase; red). At the third larval instar, expression is clearly missing from the aPA precursor, and by prepupal stages, additional “holes” corresponding to the posterior supraalar (pSA) and sensilla trichoidea 1 (tr1) precursors are obvious in the lateral notum. (A') and (B') are higher magnifications of boxed areas in (A) and (B). (C), the expression of *Toll-8* as revealed by *in situ* hybridisation with a *Toll-8* RNA probe. Expression is strong in the lateral notum. (D), thorax of a fly of the genotype *y f^36a^ abx>f^+^>Gal4; UAS Flp; Toll-8-Gal4.* All *MD806*-positive cells in these flies simultaneously express flipase which induces high levels of FRT-mediated recombination. Consequently, all bristles marked with *f^36a^* arise from cells expressing *Toll-8*. As can be seen in (3D), *f^36a^* bristles can be seen even in the medial region of the notum. (E), thorax of a *neurGal4>UAS-E(spl)m8* fly showing a complete loss of bristles. (F), thorax of *Toll-8Gal4>UAS-E(spl)m8* fly showing a full complement of bristles, indicating that *Toll-8* is not expressed in the precursors.

Genetic interactions suggested a role for Toll-8 in NF-κB/Rel regulation of the neural fate, so we examined the expression pattern of *Toll-8* in the larval wing disc. We employed *MD806*, a Gal4 insertion in the 5′UTR of *Toll-8*. *Toll-8-Gal4* drives *GFP* reporter gene expression at very high levels in the lateral halves of the notum in discs at the third larval instar (Figure 3A,B). *In situ* hybridization with a *Toll-8* probe revealed a very similar pattern of expression (Figure 3C), see also [27]. This covers the region where most of the bristles form, except the dorsocentral and scutellar bristles that arise just at the border where expression levels fall. Ectopic expression of *sc* using *Toll-8-Gal4*, resulted in the generation of ectopic bristles on the lateral notum as well as in the dorsocentral and scutellar regions (not shown). This suggests that *Toll-8* is expressed in the medial notum but that the levels there are much lower. We also used *Toll-8-Gal4* to drive *UAS*-*Flp* and obtain mitotic recombination with a cuticular marker *f^36a^* and found labelled cells over the entire notum in these flies (Figure 3D). This also indicates that *Toll-8* is expressed in the medial notum (or was expressed there earlier in development). Overall these results suggest that Toll-8 activity is likely to be higher in the lateral notum. It is noteworthy that the bristle phenotypes seen after loss or gain of function of *NF-κB/Rel* gene activity display differences between the medial and lateral notum.

Significantly, levels of transcription of *Toll-8* differ in the SOP with respect to cells surrounding it. Expression of *Toll-8,* visualized with *Toll-8-Gal4>UAS-GFP*, is gradually excluded from the SOPs as they arise (Figure 3B, B'). Exclusion from SOPs was confirmed by double labelling with the SOP reporter A101-*lacZ* (Figure 3A, A') and is particularly well illustrated by the anterior postalar (aPA), posterior supraalar (pSA) bristles and sensilla trichoidea1 (tr1) precursors in the lateral notum. In late third instar larval discs, GFP expression fades in the early arising aPA precursor, which can be seen marked in red in Figure 3A, A'. By white prepupal stages, additional “holes” in the GFP expression pattern appear at positions corresponding to the later arising pSA and tr1 precursors (Figure 3B'). Ectopic SOPs also lose *Toll-8* expression: mis-expression of *sc* using *Toll-8-Gal4* generates several ectopic cells positive for the neuronal marker A101-*lacZ*, all of which lose GFP expression (not shown). Furthermore, whereas mis-expression of *E(spl)m8* using the SOP-specific *neur-Gal4* driver results in a complete loss of notal bristles (Figure 3E), mis-expression with *Toll-8-Gal4* is without effect (Figure 3F), reinforcing the observation that *Toll-8* expression is not retained within SOPs. Exclusion of Toll-8 from neural precursors in the embryo has been previously reported [20].

Expression of *Toll-8,* visualized in *Toll-8-Gal4>UAS-GFP* flies, is retained in the epithelium after pupariation when all of the SOPs for the large bristles have formed (not shown). The significance of this, as discussed below, may lie in the fact that a further round of neurogenesis takes place some hours later, when precursors of the small bristles arise.

Expression of *Toll-8* is non-uniform in the disc epithelium and transcription is down regulated in the SOPs. If Toll-8 is affecting the activity of one or more NF-κB/Rel proteins, this would suggest, by extrapolation, a similar discontinuity between the levels of NF-κB/Rel proteins in the SOP compared to the cells surrounding it.

### NF-κB/Rel might recruited to the α boxes in the *scute* sensory organ precursor enhancer

**Figure 4:**
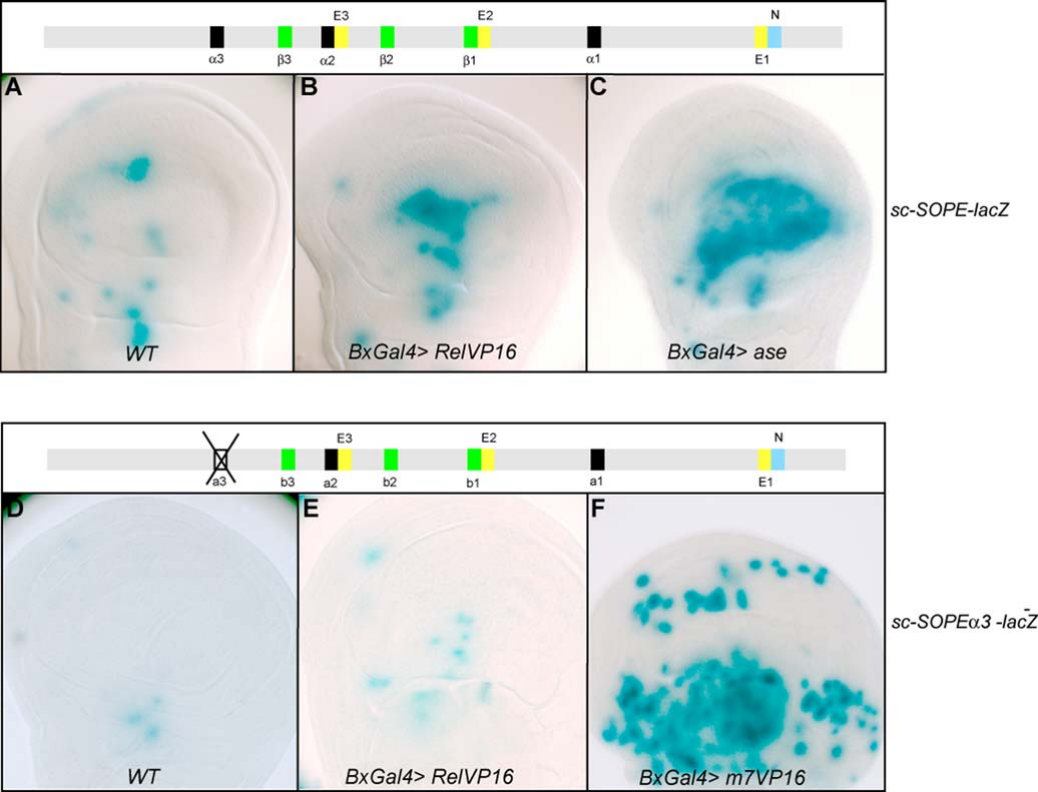
A protein composed of a fusion of Relish and VP16 can bind to the *scute* SOP-enhancer and activate transcription *in vivo.* (A–C), expression of *sc-SOPE-lacZ* in wild type (A), *Bx-Gal4>UAS-RelVP16* (B) and *Bx-Gal4>UAS-ase* discs (C). Over-expression of *asense* results in activation of the reporter gene over a larger area than over-expression of *RelVP16*. (D–F), expression of *sc-SOPEα3^−^-lacZ* (a version of the enhancer construct in which one of the NF-κB/Rel binding sites, *α3*, has been mutated) in wild type (D), *Bx-Gal4>UAS-RelVP16* (E) and *Bx-Gal4>UAS-E(spl)m7VP16* (F) discs. Expression is decreased in wild-type flies and after over-expression of RelVP16, but strongly increased in the presence of E(spl)m7VP16 whose activity does not rely on the same binding sites.

Is *sc* a direct target of NF-κB/Rel signalling? The SOP enhancer of *sc* (*sc-SOPE*) [2] contains three consensus binding sites, α boxes, for NF-κB/Rel proteins (upper cartoon in Figure 4). To examine NF-κB/Rel-mediated transcriptional regulation of *sc* we employed *sc-SOPE-lacZ,* a construct containing the native *SOPE* (called *SRV-lacZ* in [2]) and *sc-SOPEα3^−^-lacZ,* a construct in which one of the α boxes, α3, has been mutated. In wild-type discs *sc-SOPE-lacZ* is expressed in all SOPs (Fig 4A), but expression of *sc-SOPEα3^−^-lacZ* is eliminated in all but four cells (Figure 4D) suggesting this motif is essential for activation [2].

To test for a direct role of NF-κB/Rel we expressed a chimeric protein containing full length Relish fused in-frame to the *trans-*activation domain of VP16, a potent transcriptional activator. The *Gal4>UAS* system was used to express Rel-VP16, but note that signalling is nevertheless required to process Rel-VP16 and allow nuclear access. We used *Bx-Gal4,* which drives expression in the wing pouch (visualized in Figure 4F, see below). The *Bx* expression domain overlaps an area of *Toll-8* expression as well as proneural clusters of *ac-sc* expression from which sensilla of the wing margin, dorsal radius and third wing vein arise. *sc-SOPE-lacZ* staining was increased in *Bx-Gal4>UAS-Rel-VP16* wing discs in a region in the centre of the *Bx-Gal4* expression domain (Figure 4B). More cells are labelled than in wild-type discs (Figure 4A). In contrast, *sc-SOPEα3^−^-lacZ* is only very weakly expressed in *Bx-Gal4>UAS-Rel-VP16* animals, although it can still be detected in more cells than in the control discs (Figure 4D,E). Therefore Rel-VP16 cannot efficiently activate ectopic transcription of the SOPE in the absence of the α3 site.

As a control for this experiment we employed a chimeric protein comprised of E(spl)m7, a transcription factor that would bind the intact E or N boxes in *sc-SOPEα3^−^-lacZ*, and the activator VP16 [28]. Expression of this protein using *Bx-Gal4* results in dramatic expression of *sc-SOPEα3^−^-lacZ* in most cells of the *Bx* expression domain (Figure 4F).

We noted that the ectopic cells with *sc-SOPE-lacZ* staining in *Bx-Gal4>UAS-Rel-VP16* flies were close to the positions of extant SOPs (seen in Figure 4A) suggesting that they have arisen from cells of the proneural clusters originally expressing *ac-sc* (Figure 4B). In contrast, when the same *Bx-Gal4* driver is used to ectopically express Ase, a proneural transcription factor that would bind the E boxes in *sc-SOPE-lacZ*, a dramatic up-regulation in many more cells throughout much of the *Bx* expression domain is seen (Figure 4C) These include cells outside the areas of *ac-sc* expression in the wild type. These results suggest that Rel-VP16 can only activate the SOPE in the cells that have high levels of Ac/Sc.

We suggest that NF-κB/Rel proteins might be recruited to the *sc* SOP enhancer via the appropriate α binding sites and could therefore directly activate or repress *sc* expression. Activation in the sensory organ precursor may require high levels of Ac/Sc.

### *scute*, *asense* and *senseless* are ectopically transcribed in *Relish* mutants

*Toll-8* is expressed throughout the epithelium and is excluded from SOPs. Further, loss-of-function mutants in *Toll-8* and the NF-κB/Rel-encoding genes bear ectopic bristles. We therefore examined expression of the neuronal genes *sc, ase* and *sens* in these mutants. We chose to examine *Toll-8^1^* and *Rel^E20^* mutant discs because of the strong phenotype of *Rel^E20^* and the genetic interaction between these two mutants.

**Figure 5:**
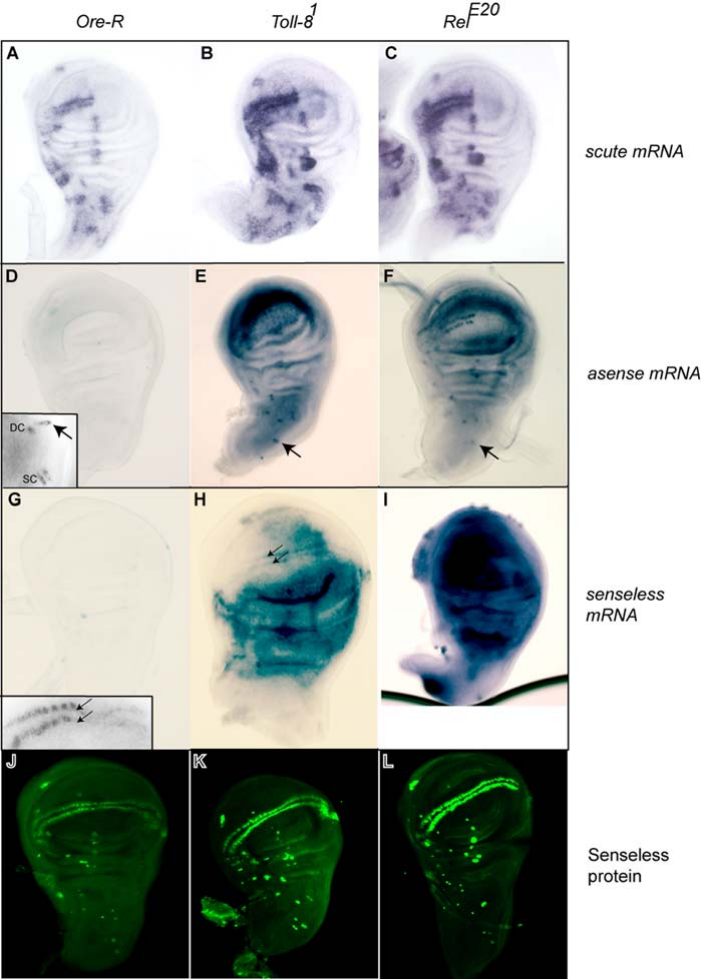
Expression of neuronal genes in *Toll-8* and *Relish* mutants. (A–I), *in situ* hybridisation with probes for *scute, asense* and *senseless* in wild type and in *Toll-8^1^* and *Rel^E20^* mutant discs. Each set of three discs was processed in the same way and stained for the same length of time. *scute* transcripts are present at higher levels in the proneural clusters and the clusters themselves appear enlarged (A–C). Expression of *asense* (D–F) and *senseless* (G–I) is also much stronger in the mutant discs in bristle precursors, the cells to which they are confined in the wild type. Staining in the mutants was already strong before any staining in the wild type had become visible. Arrows in (E) and (F) point to the precursors arising from the dorsocentral cluster and insets in (D) and (G) show SOPs in wild-type discs stained for 40 minutes. In addition, *asense* and *senseless* transcripts can be seen to accumulate ectopically over most of the epithelium at very high levels. (J–L), staining with an antibody against Senseless. The protein is present only in sensory organ precursors.

Two surprising features were observed. First, *sc*, *ase* and *sens* were seen to be expressed at higher levels in *Toll-8^1^* and *Rel^E20^* mutants than in wild-type control discs (Figure 5A–I). Samples for each genotype were fixed, processed and stained under identical conditions. Staining times were kept short to better visualize differences in staining intensity. Typically, within 5 minutes of staining, *ase* expression was apparent in the SOP cells of mutant discs, but not the wild-type controls. Second, the neuronal genes were expressed in significantly broader domains in the mutants. *scute* was seen to be expressed in enlarged proneural clusters (Figure 5A–C). *asense* and *sens,* whose expression is usually confined to the SOP, were globally de-repressed in the mutants and transcripts for these genes accumulated over much of the disc (Figure 5D–I). This is unexpected because only a few ectopic bristles develop in these mutants and ectopic expression of Ase and Sens in wild-type discs results in the generation of vast numbers of ectopic bristles. However, the ectopic transcripts were not mirrored by a corresponding accumulation of the protein products of these genes. We found that Sens protein is restricted to the SOP cells (Figure 5J–L). It appears that the de-repressed *ase* transcripts in *Toll-8^1^* and *Rel^E20^* mutants are not translated either (see Figure S2A–C). The distribution of other neuronal markers such as Achaete (Ac) (Figure S2D–F) or Hindsight (not shown) was also confined to the precursors in *Toll-8^1^* and *Rel^E20^* mutants.

We conclude that transcripts of *sc, ase* and *sens* are present both ectopically and at higher levels in *Toll-8^1^* and *Rel^E20^* mutants. The transcripts are not translated, however, and therefore do not result in the formation of large numbers of ectopic bristles.

### The de-repression of target genes in *Relish* mutants is mediated by post-transcriptional effects on mRNA stability

To investigate whether the increased levels of *sc, ase* and *sens* mRNA present in mutant *Rel^E20^* discs are a result of increased transcription or greater transcript stability, we used a heterologous expression system based on *Gal4>UAS* activation. A recombinant chromosome bearing *sca-Gal4* and *UAS-GFP* was placed in *trans* with chromosomes bearing *UAS-ac, UAS-sc* or *UAS-ase*, either with or without functional copies of the *Rel* gene. If the increase in mRNA in the *Rel* mutant is due to increased transcription, then no increase should be possible from a heterologous promoter. If, on the other hand, the transcripts are stabilized then mRNA from the heterologous promoter should accumulate in this experiment.

**Figure 6:**
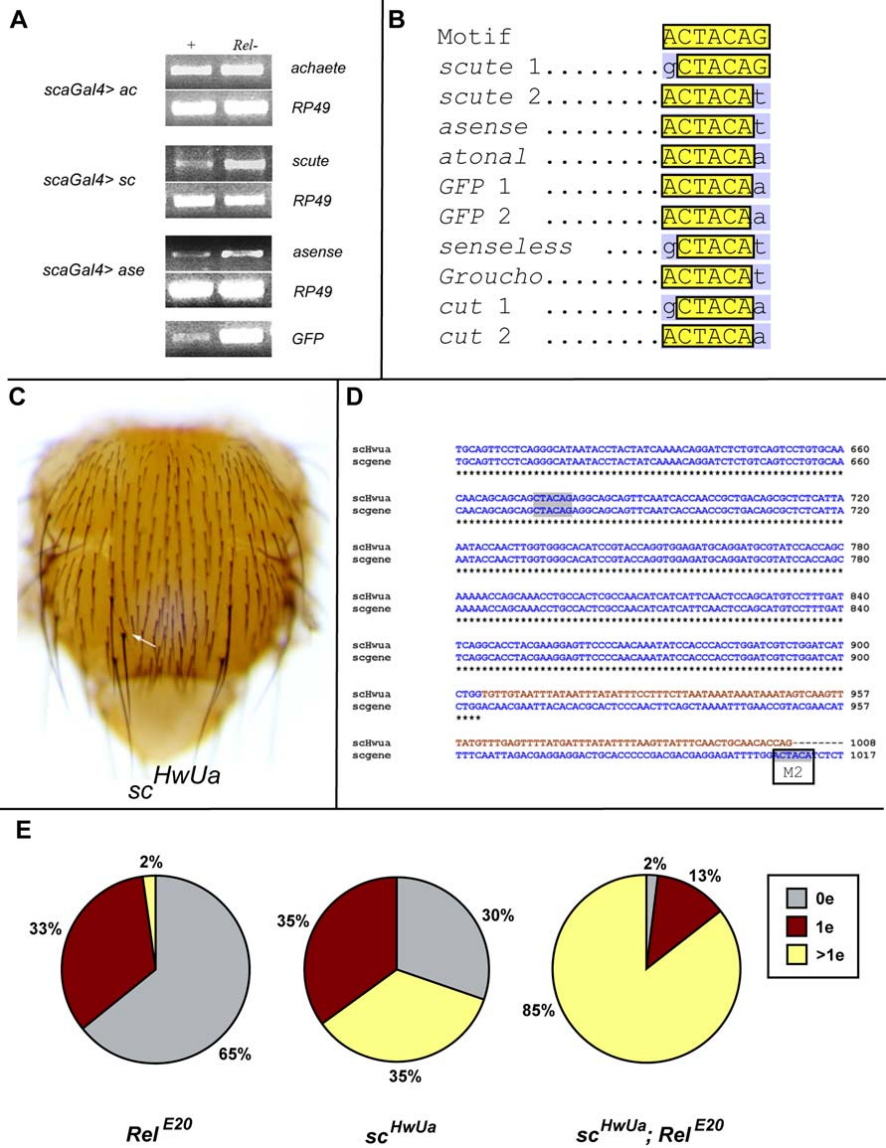
Transcript stability in *Relish* mutants correlates with the presence of a heptamer sequence in the coding regions of target genes. (A), levels of heterologous (UAS) *achaete, scute, asense* and *GFP* transcripts driven by *sca*-Gal4 in wild-type (left column) and *Rel^E20^* (right column) flies. The ribosomal RNA RP49 was used as a loading control. Levels of *scute* and *asense* but not *ac* transcripts are elevated in the mutant. In addition levels of GFP transcript are elevated. (B), a sequence motif similar to that described in *Sox9* and *MyoD* (ACTAGA) is present in *scute, asense, senseless* and a number of other genes involved in patterning the notum. The central five core element nucleotides, CTACA, are conserved in all cases. The 3′-most nucleotide, G, is replaced in most cases by a T or an A. The 5′-most nucleotide, A, is replaced by a G in three cases (shown in grey). The final base, G, is mostly substituted by A or T. (C), the sequence of *scHw^Ua^* is presented. Transcription stops within the *copia* element whose sequence is given in red. The two *MyoD* motifs are outlined in grey; one, M2, is predicted to be absent from the truncated transcript. (D), a photograph of a *scHw^Ua^* mutant fly showing the presence of an ectopic dorsocentral bristle (white arrow). (E), pie charts representing the percentage of heminota displaying ectopic bristles from a total of 200. Red sectors indicate the percentage with one ectopic macrochaete, yellow sectors two or more ectopic macrochaetes (p>0.001 when compared with wild type).

Reverse primers located within the 3′UTR of the ectopic transcripts (see Experimental Procedures) were used to prime cDNA synthesis and detect transcript levels. Transcripts specific for *ac* are not present at higher levels in the mutant, suggesting that this gene is not subject to regulation by NF-κB/Rel (Figure 6A). This result also indicates that loss of *Rel* activity does not have a significant impact on the expression of *sca-Gal4*. We detected enhanced levels of *sc* and *ase* transcripts in the *Rel^E20^* homozygous mutant background (Figure 6A). Unexpectedly, *GFP* transcripts were also present at much higher levels in the mutant (Figure 6A). *GFP* transcripts similarly result exclusively from activity of the *Gal4>UAS* driver and therefore cannot be subject to direct transcriptional regulation by Relish. We conclude that stability of the ectopic *GFP, sc* and *ase* mRNAs is increased in the mutant.

It has been shown that NF-κB can regulate target gene expression using post-transcriptional as well as transcriptional mechanisms. Loss of NF-κB signalling causes a increase in endogenous mRNA levels of *MyoD* and *Sox9* in C2C12 cells, an effect dependent on a heptamer motif, ACTACAG, present in the coding sequence of both genes [17]. Examination of the coding sequences of *sc, ase* and *GFP* reveals the presence of a similar, but slightly modified motif, in which six of the seven nucleotides are conserved (ACTACA- Figure 6B). Two copies of the motif are present in the coding region of *sc;* a single copy is present in both the *ase* and *sens* genes. Notably the motif is present twice in the coding sequence of *GFP*. It is also present in other genes involved in patterning the notum (Figure 6B). Interestingly this sequence is not found in *ac*, a fact consistent with the lack of transcript accumulation in *ac* mutants.

If the heptamer motif does indeed mediate rapid turnover of *sc, ase* and *sens* transcripts, then mRNA of mutants devoid of this motif should be stabilized. Examination of existing *sc* mutants led us to one such mutant: *scHw^Ua^*. *scHw^Ua^* carries a complete *copia* element within the *sc* coding sequence causing a truncated transcript [29]. We have located the *copia* insertion to position 904bp within the *sc* gene near the end of the coding region. The aberrant transcripts would therefore retain only one of the heptamer motifs (Site M2, Figure 6D). Interestingly, *scHw^Ua^* mutant flies display one or two ectopic bristles (Figure 6C,E) and furthermore a 5-fold increase in *sc* transcript levels [29]. If Relish acts via the heptamer motif, then double mutant *scHw^Ua^ Rel^E20^* flies may be expected to display a more extreme phenotype than that of *scHw^Ua^*. Indeed *scHw^Ua^ Rel^E20^* flies have more bristles than *scHw^Ua^ Rel^+^* animals (Figure 6E). *scHw^Ua^ Rel^E20^* flies also have more bristles than *Rel^E20^* flies suggesting that Relish is not the only NF-κB/Rel factor involved in this phenotype. These results suggest a possible role of the heptamer sequence in transcript stability.

We conclude that Relish acts on *sc* and *ase* through a post-transcriptional mechanism inducing rapid mRNA turnover. The *scHw^Ua^* phenotype is consistent with a role for a heptamer motif in the transcribed regions of these genes that is similar to that regulating transcript stability in *MyoD* and *Sox9* [17].

## Discussion

### Dual regulation of neural genes by NF-κB/Rel

**Figure 7:**
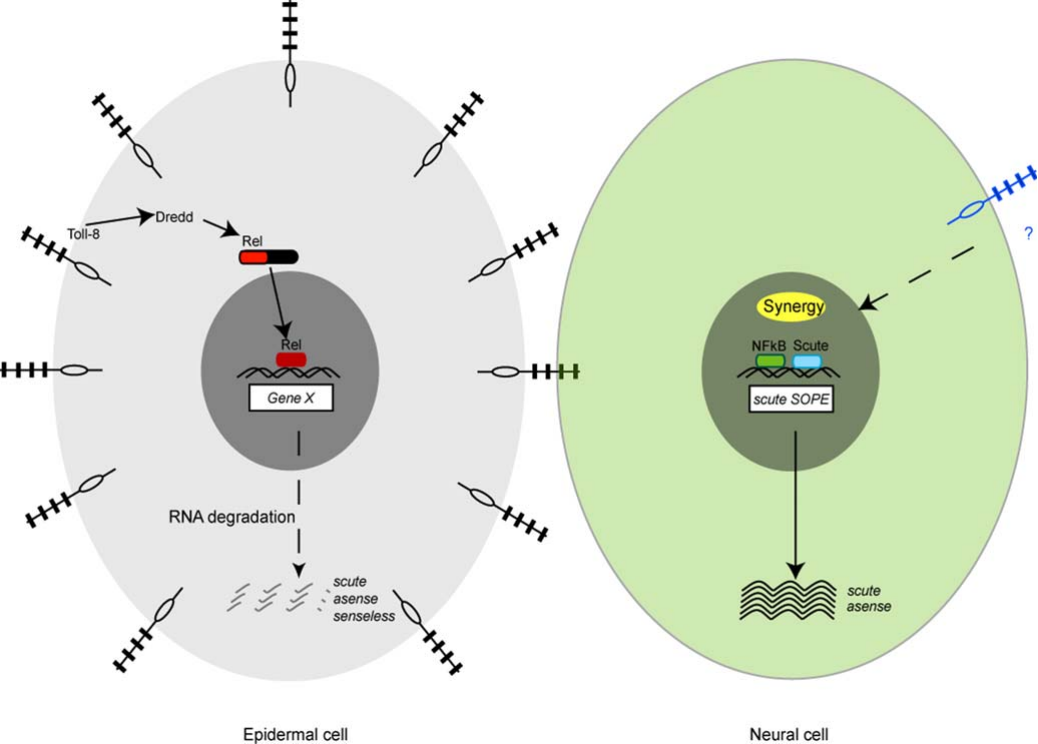
A model for the role of NF-κB proteins in buffering levels of the neural genes *scute* and *asense.* Epidermal cells express high levels of Toll-8, which promotes high nuclear levels of NF-κB/Rel. Relish activates the transcription of an unknown target gene, whose activity results in the degradation of *scute* and *asense* transcripts. This activity extends throughout the neuro-epithelium of the disc. Once mature precursors are chosen, they stop expressing Toll-8 and low levels of the NF-κB/Rel proteins might be recruited to the *scute* SOPE, where they would synergize with the bHLH protein Scute to activate transcription of high levels of *scute*.

Our results suggest a dual role for the NF-κB/Rel proteins of *Drosophila* in the formation of SOPs (Figure 7). First, they could be recruited directly to the *sc* promoter and regulate transcription. The SOP enhancer of *sc,* required for auto-regulation of *sc* in the SOPs, contains α boxes, consensus sequences for NF-κB/Rel [2]. Culi and Modolell (1998) obtained evidence for a role of these sequences in both activation and repression of *sc*. Expression of Rel-VP16, a potent transcriptional activator form of Relish, is able to ectopically activate a reporter gene containing the intact *sc* SOP enhancer but not one in which the α3 box is mutated. So activation in this experimental situation requires the presence of an intact α3 site. The experiment does not rule out indirect effects, so further work is required to verify whether activation is direct. We suggest the NF-κB/Rel proteins participate in activation and repression of transcription of *sc,* a hypothesis consistent with *dl, Dif* and *Rel* mutant phenotypes of additional as well as missing bristles. Second, we describe an unexpected role of *Rel* in mRNA turnover of *sc, ase* and *sens*, neuronal genes required to specify and/or maintain the neuronal fate of SOP cells [8], [9], [10], [11], [12], [13]. In *Rel* mutants, transcripts of *sc, ase* and *sens* accumulate due to increased transcript stability. Therefore in the wild type, Relish promotes rapid mRNA turnover, presumably indirectly through an unidentified transcriptional target. A similar phenotype is observed in *Toll-8* mutants, which furthermore, interact genetically with *Rel* mutants. Transcripts for *Rel* are reduced in the *Toll-8* mutant suggesting a role for Toll-8 in maintaining the levels of *Rel* transcript (Figure S3). This might be the reason for the genetic interaction.

A number of differences are apparent between mutants of the three NF-κB/Rel-encoding genes of *Drosophila*. Mutants triply homo- or hetero-zygous have a normal complement of bristles, while single homo- or hetero-zygous animals have either additional or missing bristles. This suggests possible opposing functions for these genes. Furthermore bristle phenotypes due to loss or gain of function differ in detail between the three mutants. Together these results point to the importance of the stoichiometric relationships between the three NF-κB/Rel proteins and raise the possibility that different Dorsal/Dif/Relish homo- or hetero-dimers may have distinct binding sites and therefore different targets [30], [31], [32]. This merits further investigation.

### The ratio of proneural to NF-κB/Rel proteins might determine activation or repression of transcription of *scute*

If NF-κB/Rel proteins both activate and repress *sc,* then they are expected to activate in SOP cells and repress in cells of the proneural clusters not chosen to be SOPs. We discuss two possible ways that this could occur. First, activation in the SOP may rely on high levels of proneural protein and low levels of *NF-κB/Rel* protein; conversely repression may require low levels of proneural and high levels of *NF-κB/Rel* protein. Notch-mediated lateral inhibition results in high levels of Sc in the SOP and lower levels in surrounding cells. *Toll-8* expression is excluded from SOP cells suggesting, that, if Toll-8 affects NF-κB/Rel activity, there would be lower levels of NF-κB/Rel in SOPs. NF-κB has been shown to activate transcription even without stimulus if IκB levels are low enough to allow NF-κB-dependent gene expression in the basal state [33]. Interestingly, it has been shown that low levels of Dorsal can act synergistically with bHLH proteins to activate target genes in the embryo [34]. This depends on direct association of Dorsal and bHLH proteins and cooperative binding to closely linked binding sites for the two respective proteins [34], [35]. Furthermore these authors demonstrated cooperative binding for Sc and Dorsal. In the *sc* SOP enhancer one of the α boxes is indeed close to an E box, so perhaps high levels of Sc and low levels of NF-κB/Rel combine to activate transcription in the SOP. Two observations are consistent with this hypothesis: Rel-VP16 was able to ectopically activate *sc-SOPE-lacZ* only at sites where *ac* and *sc* are expressed and, after over-expression of NF-κB/Rel proteins, bristles are generally missing on the lateral notum (where Toll-8 levels are high), whereas ectopic bristles are found on the medial notum (where Toll-8 levels are low).

A second means by which NF-κB/Rel proteins could act differently in SOP and in non-SOP cells, may be the presence/absence of co-factors. It has been shown that Dorsal can be converted from an activator to a repressor by association with the co-repressor Groucho [36]. This bi-functionality is attributable to the fact that Dl only weakly interacts with Gro [37]. During embryogenesis both Cut and Deadringer bind an AT-rich silencer sequence, AT2, present in target genes of Dorsal and both Dorsal and Deadringer bind the co-repressor Groucho and recruit it to DNA [38]. A similar AT-rich sequence (the β box) is present in the *sc* SOP enhancer [2]. Furthermore repression of *sc* by the *E(spl)* proteins, targets of Notch signalling in non-SOP cells, is already known to require the activity of Groucho [2], [39], [40], [41].

### Toll-8 and Relish promote rapid turnover of transcripts of neuronal genes

Transcripts for *sc, ase* and *sens* (and GFP) accumulate in *Rel* and *Toll-8* mutants as a result of increased transcript stability. Transcript stability correlates with the presence of a six or seven nucleotide motif in the transcribed sequence of these genes. The motif is present in *sc, ase* and *sens,* but not *ac* the transcription of which is unaffected in *Rel* mutants. The motif is almost identical to the heptamer in *MyoD* and *Sox9* that is associated with transcript stability after inhibition of NF-κB/Rel signalling in C2C12 cells [17]. A *sc* mutant with a truncated *sc* transcript lacking one of the two motifs present in the coding sequence of this gene, has a phenotype similar to *Rel* and *Toll-8* mutants and an increase in *sc* mRNA. Rabinow et al (1993) suggested that increased stability of the transcripts rather than increased transcription underlies this phenotype. We note the presence of the heptamer in a number of genes involved in sensory organ patterning suggesting possible regulation by NF-κB/Rel of a battery of genes in the imaginal epithelium. A similar motif is present in other vertebrate targets of NF-κB/Rel [42]. Post-transcriptional regulation of target genes by NF-κB/Rel could therefore be an ancient feature common to *Drosophila* and mammals and possibly even jellyfish. Sitcheran et al [17] suggest that an unknown factor, presumably a transcriptional target of NF-κB/Rel, regulates messenger turnover through association with this sequence. In *Rel* and *Toll-8* mutants the accumulated transcripts are not translated. This must be an effect of the mutants because ectopic expression in wild-type flies allows translation and ectopic bristle formation.

Promotion of a rapid turnover of transcripts of neuronal genes presumably does not take place in the SOPs where high levels of the protein products of these genes are required. Accordingly *Toll-8* expression is extinguished in the SOPs after their formation. Factors specific to the SOP presumably allow translation of the transcripts. We therefore suggest that high levels of Relish provided by Toll-8 in non-SOP cells might be required for post-transcriptional regulation of neuronal genes.

### Maintenance of steady state levels of gene expression by NFκB/Rel may keep the neuro-epithelium primed for neurogenesis

In wild-type animals expression of neuronal precursor genes such as *sens* and *ase* is restricted to SOPs where they are activated by high levels of Ac and Sc [9], [10], [11]. Our results suggest that they are in fact expressed over the entire neuro-epithelium but that mRNA turnover is rapid due to NF-κB/Rel activity. Activation of *ac-sc* in proneural clusters would counteract the effects of NF-κB/Rel to allow selection of SOPs. After selection of SOPs for the large sensory bristles is finished, *Toll-8* expression is maintained in the epithelium, suggesting that high levels of NF-κB/Rel are still required for continued transcript turnover. Continuous buffering of neuronal gene expression presumably continues until the next round of neurogenesis that takes place after pupariation when precursors for the small bristles form. Therefore we hypothesize that NF-κB/Rel plays a subtle role in maintaining steady state levels of expression of many genes required for neural development. The maintenance of low levels of expression of neuronal genes would keep the tissue poised for neurogenesis that takes place in repeated rounds. Perhaps low levels of expression of neuronal genes are characteristic of neuro-epithelia in general.

### Conclusions

Our hypothesis concerning the dual role of NF-κB/Rel in neurogenesis in *Drosophila* is as follows. The neuro-epithelium of the imaginal discs expresses neuronal genes. Prior to development of SOPs, high levels of Toll-8 maintain high levels of Rel and result in nuclear accumulation of NF-κB/Rel. Through an unknown transcriptional target(s), Relish promotes rapid turnover of neuronal transcripts by a post-transcriptional mechanism. This might be mediated by a specific sequence in the coding regions of target genes. Activation of *ac* and *sc* in proneural clusters by regulatory proteins of the notal prepattern counteracts the effects of Relish. After singling out of SOPs by Notch-mediated lateral inhibition, *Toll-8* expression ceases in the SOPs. Reduced levels of signal uncover a *trans-*activator function for NF-κB/Rel that, synergistically with Sc, helps to maintain high levels of *sc* expression in the SOP, possibly through direct binding to consensus sequences in the *sc* SOP enhancer. The NF-κB/Rel proteins may also directly repress *sc* in non-SOP cells of the proneural clusters. It remains to be seen to what extent each of the three proteins participates in these two processes.

## Materials and Methods

### *Drosophila* culture and stocks

Flies were maintained on standard cornmeal-agar medium at 18°C and Oregon-R was used as a control. Strains used were: dl^1^ cn sca^1^/Cyo, dl^4^ pr1 cn1 wxwxt bw1/CyO, Dif^1^ cn bw/CyO, Df(2L)J4/Cyo-GFP, Df(2L)TW119/Cyo, Rel^E20^/TM6b (other alleles of Rel (Rel^F13^, Rel^F40^ and Rel^KG^ display a similar phenotype, not shown), Toll-8^1^/TM6b, Dredd^EP1412^, sc[HwUa] and sc[HwUa]; Rel^E20^. Strains used for NFκB/Rel misexpression were sca[537.4]Gal4>GFP, Bx[MS1096] Gal4, pnr[MD237]Gal4>GFP, Mae-UAS.6.11-dl^UY2278^, Mae-UAS.6.11-Dif^LA00958^, UAS-HA-Rel and UAS-HA-VP16-Rel. lacZ reporter strains used were sc-SOPE-lacZ, sc-SOPEα3^−^-lacZ and neur[A101]-lacZ. Other strains used for ectopic expression were UAS-E(spl)m8, UAS-E(spl)m7ACT, UAS-ac, UAS-sc and UAS-HAase. The genotype y f^36a^ abx>f^+^>Gal4; UAS Flp; Toll-8-Gal4 was used to ascertain the extent of Toll-8 expression. See FlyBase for a description of mutants (http://flybase.bio.indiana.edu/).

UAS-constructs for ectopic expression of haemagglutin (HA)-tagged Rel were generated by standard techniques. The VP16 TA domain was PCR-amplified from a fly bearing the Ubx-VP16 fusion, and cloned in frame into pHA-Rel to generate an N-terminal HA-VP16-Rel fusion.

### Mutagenesis

The fly strain MD806 [21] was identified as an insertion in the 5′-UTR of *Toll-8* by plasmid rescue and used to generate the *Toll-8^1^* deletion by standard P-element excision. Several P[w-] strains were established and the extents of the deletions were confirmed by PCR. Primer pair U6 (CTCAGCCACCGCCACCTCAT) and L10 (GGTGACAAGCGGAGAGCATTG) was used to determine the precise breakpoints of the R5A strain and primer pair U5 (AGCCCTCAGCAAGACGGTG) and L5 (AAGATTCCTGGGGGCCAGTAC) was used to generate a probe for *in situ* hybridization.

### Bristle scoring

All mutant chromosomes used for the scoring were placed over either *CyO-GFP* (Chr II) or *TM6b,Tb* (Chr III) chromosomes and animals of the appropriate genotypes were selected as Non-GFP or Non-*Tb* individuals. Triple null animals were selected as Non-GFP, Non-*Tb* animals and confirmed by single-fly genomic PCR using primers specific for *dorsal (*DLf1 AGGGTCCAGCAGTTGATG, DLr1 TGCTTGTGGACATCCGTG), *Dif (*DIFF1 CCAGCATGGAGTTGAATGG, DIFR1 GATCTCGGTGTTCCTGTAG*)*, *Rel* (Rel5 CCAACCTTAATCTCCGAG, Rel9 AATATGCGTGTGCGAGCG). The unrelated third chromosome gene *delilah* (Dei1 GATCTGAATGACATGGCC, Dei2 CGGCCTGTATTAGTTCGT) was used as an independent control.

Females grown at 18°C were examined for ectopic bristles and 200 hemithoraces were scored for each genotype, except *dl^4^/dl^1^* (146 hemithoraces). Statistical analysis was performed using Student's T-test. Pie charts were generated using Microsoft Excel.

### Histochemistry and immunolabelling

Primary antibodies used included Anti-GFP-Alexa 488 (Molecular Probes), anti-Senseless (Bellen lab), anti-βgal 40-1a, anti-Achaete and Anti-Hindsight (DSHB). Secondary antibodies coupled to Alexa-488, Alexa-546 and Alexa-647 (Molecular Probes) were used. Fluorescence images were taken with a Leica microscope using FW4000 software. Images were processed with Adobe Photoshop.

### *In situ* hybridization

Samples were fixed overnight in 4% formaldehyde and then processed for *in situ* hybridization using standard techniques. Full-length cDNA clones of *sc* and *ase* and a 1kb PCR-amplified fragment of *sens* were used as templates to generate DIG-labelled RNA probes. All samples were processed in identical fashion. The experiments were repeated at least 4 times.

### Ectopic expression assay

*sca[537.4]>GFP*, *UAS-ac, UAS-sc* and *UAS-HA-ase* second-chromosome insert stocks were established individually with *Rel^E20^* on the third chromosome. *Gal4>UAS* crosses were set up simultaneously as the *Gal4>UAS; Rel^E20^* crosses. 10 wandering larvae were collected for each pair and processed for semi-quantitative RT-PCR. RNA was extracted using TRIzol reagent (Life Technologies) and 200ng of this RNA was used for reverse transcription using gene-specific reverse primers and SuperScript™ II RNAseH^−^ Reverse Transcriptase (Life Technologies). Total RNA was treated with DNAse I to remove possible contaminating genomic DNA. Transcripts of the ribosomal protein RP49 were used as RNA loading controls. PCR amplification using gene-specific forward and reverse primers (25 cycles) was performed using Taq DNA polymerase (Roche) on an Eppendorf Mastercycler. Since the amplified transcripts represented a mixture of endogenous and ectopic RNA, we repeated the reverse priming with primers located in the SV40 tail of the UAS constructs. Subsequent PCR using gene-specific primers then resulted in the exclusive amplification of ectopic (UAS) transcripts.

Primers used were SV401 CCGGTAGGTAGTTTGTCC, SV402 GGGGCCTTCACAAAGATC, RP49-5′ ATGACCATCCGCCCAGCATAC, RP49-3′ TTACCTCGTTCTTCTTGAGAC, Acf1 GCTTGCAGAAAGTTCTTCATG, Acr1 GTTTTTTTCAGGTCGTCCTG, Scf1 CCATGTCATCGAGTGTGC, Scr1 ACTGTGACTGCTGGACTC, Asef3 GGCACAACCAGCAGAATC, Aser3 CTTCTTGAATCCGGGAAG, Gfp1 AGGAGAAGAACTTTTCACTG, Gfp2 CCCTTGTTAATAGAATCGAG

The *scute* gene from the *HwUa* allele was amplified using primers Scf1 (CCATGTCATCGAGTGTGC) and Copiar1 (GTGCTGGTGTTGCAGTTG). This PCR fragment was sequenced and the exact breakpoint of the *copia* insertion was established.

## Supporting Information

Figure S1 Recovery of a null allele of *Toll-8*. The insertion in MD806 maps 160bp upstream of the site of initiation of transcription and 640bp upstream of the translation start site and so was used to generate *Toll-8* mutants by imprecise excision (A), several mutant lines were established and the extents of the deletions were confirmed by genomic PCR. Several primer pairs spanning the length of the *Toll-8* gene and its upstream sequences were used to test the R5A strain and a 1.8kb U6L10 fragment normally spanning 4kb in wild-type flies was cloned and sequenced to confirm the extent of the deletion. This mutant strain was found to have a deletion of 2.24kb in the *Toll-8* gene and was renamed *Toll-8^1^*. (B), *Toll-8^1^* flies lack detectable transcript as judged by *in situ* hybridization with a U5L5 probe (C) and are predicted to be protein-null due to the absence of the usual translational start site.
(0.29 MB TIF)

Figure S2 The protein products of neuronal precursor genes are confined to sensory organ precursors in *Toll-8* and *Relish* mutants. The neuronal-specific reporter gene *sc-SOPE, lacZ* is found in a normal complement of precursors in wild type (A), *Toll-8^1^* (B) and *Rel^E20^* (C) mutants. *sc-SOPE-lacZ* contains three E-boxes, which are binding sites for bHLH proteins such as Scute and Asense. Over-expression of ase in wild-type animals leads to ectopic expression of *sc-SOPE-lacZ*, presumably due to generation of functional Ase protein (see Figure 3C). However, the ectopic expression of ase observed in the NF-κB mutants does not generate a corresponding global overexpression of *sc-SOPE-lacZ* (A–C), leading to the conclusion that Ase function in these discs is still confined to the SOPs. Staining with an antibody against Achaete shows a pattern of expression in the normal numbers and positions of the bristle precursors in wild type (D), *Toll-8^1^* (E) and *Rel^E20^* (F) mutants.
(0.63 MB TIF)

Figure S3 Toll-8 is required to maintain transcription of *Relish.Relish* transcripts are reduced in *Toll-8^1^* homozygotes (A), so Toll-8 may affect transcription of *Relish*. However, over-expression of Toll-8 in *Gal4-C765>UAS-Toll-8* does not lead to a concomitant elevation in levels of *Relish* transcripts (B), indicating that the role of Toll-8 may be confined to maintenance of *Relish* transcript levels.
(0.08 MB TIF)

## References

[1] Bertrand N, Castro DS, Guillemot F (2002). Proneural genes and the specification of neural cell types.. Nature Reviews Neuroscience.

[2] Culi J, Modolell J (1998). Proneural gene self-stimulation in neural precursors: an essential mechanism for sense organ development that is regulated by Notch signaling.. Genes Dev.

[3] Calleja M, Renaud O, Usui K, Pistillo D, Morata G (2002). How to pattern an epithelium: lessons from achaete-scute regulation on the notum of Drosophila.. Gene.

[4] Cubas P, de Celis JF, Campuzano S, Modolell J (1991). Proneural clusters of *achaete-scute* expression and the generation of sensory organs in the *Drosophila* imaginal wing disc.. GenesDev.

[5] Skeath JB, Carroll SB (1991). Regulation of *achaete-scute* gene expression and sensory organ pattern formation in the *Drosophila* wing.. Genes Dev.

[6] Heitzler P, Simpson P (1991). The choice of cell fate in the epidermis of *Drosophila*.. Cell.

[7] Brand M, Jarman AP, Jan LY, Jan YN (1993). asense is a Drosophila neural precursor gene and is capable of initiating sense organ formation.. Development.

[8] Dominguez M, Campuzano S (1993). asense, a member of the Drosophila achaete-scute complex, is a proneural and neural differentiation gene.. Embo J.

[9] Gonzalez F, Romani S, Cubas P, Modolell J, Campuzano S (1989). Molecular analysis of the *asense* gene, a member of the *achaete-scute* complex of *Drosophila melanogaster*, and its novel role in optic lobe development.. Embo J.

[10] Jarman AP, Brand M, Jan LY, Jan YN (1993). The regulation and function of the helix-loop-helix gene, asense, in Drosophila neural precursors.. Development.

[11] Nolo R, Abbott LA, Bellen HJ (2000). Senseless, a Zn finger transcription factor, is necessary and sufficient for sensory organ development in Drosophila.. Cell.

[12] Jafar-Nejad H, Acar M, Nolo R, Lacin H, Pan H (2003). Senseless acts as a binary switch during sensory organ precursor selection.. Genes Dev.

[13] Lai EC (2003). Drosophila tufted is a gain-of-function allele of the proneural gene amos.. Genetics.

[14] Royet J, Reichhart JM, Hoffmann JA (2005). Sensing and signaling during infection in Drosophila.. Curr Opin Immunol.

[15] Moussian B, Roth S (2005). Dorsoventral axis formation in the Drosophila embryo–shaping and transducing a morphogen gradient.. Curr Biol.

[16] Lemaitre B, Nicolas E, Michaut L, Reichhart JM, Hoffmann JA (1996). The dorsoventral regulatory gene cassette spatzle/Toll/cactus controls the potent antifungal response in Drosophila adults.. Cell.

[17] Sitcheran R, Cogswell PC, Baldwin AS (2003). NF-kappaB mediates inhibition of mesenchymal cell differentiation through a posttranscriptional gene silencing mechanism.. Genes Dev.

[18] Cantera R, Roos E, Engstrom Y (1999). Dif and cactus are colocalized in the larval nervous system of Drosophila melanogaster.. J Neurobiol.

[19] Kambris Z, Hoffmann JA, Imler JL, Capovilla M (2002). Tissue and stage-specific expression of the Tolls in Drosophila embryos.. Gene Expr Patterns.

[20] Seppo A, Matani P, Sharrow M, Tiemeyer M (2003). Induction of neuron-specific glycosylation by Tollo/Toll-8, a Drosophila Toll-like receptor expressed in non-neural cells.. Development.

[21] Calleja M, Moreno E, Pelaz S, Morata G (1996). Visualization of gene expression in living adult *Drosophila*.. Science.

[22] Stein D, Goltz JS, Jurcsak J, Stevens L (1998). The Dorsal-related immunity factor (Dif) can define the dorsal-ventral axis of polarity in the Drosophila embryo.. Development.

[23] Wu LP, Anderson KV (1998). Regulated nuclear import of Rel proteins in the Drosophila immune response.. Nature.

[24] Dushay MS, Asling B, Hultmark D (1996). Origins of immunity: Relish, a compound Rel-like gene in the antibacterial defense of Drosophila.. Proc Natl Acad Sci U S A.

[25] Stoven S, Silverman N, Junell A, Hedengren-Olcott M, Erturk D (2003). Caspase-mediated processing of the Drosophila NF-kappaB factor Relish.. Proc Natl Acad Sci U S A.

[26] Leulier F, Rodriguez A, Khush RS, Abrams JM, Lemaitre B (2000). The Drosophila caspase Dredd is required to resist gram-negative bacterial infection.. EMBO Rep.

[27] Kim S, Chung S, Yoon J, Choi KW, Yim J (2006). Ectopic expression of Tollo/Toll-8 antagonizes Dpp signaling and induces cell sorting in the Drosophila wing.. Genesis.

[28] Jimenez G, Ish-Horowicz D (1997). A chimeric enhancer-of-split transcriptional activator drives neural development and achaete-scute expression.. Mol Cell Biol.

[29] Campuzano S, Balcells L, Villares R, Carramolino L, Garcia-Alonso L (1986). Excess function hairy-wing mutations caused by gypsy and copia insertions within structural genes of the achaete-scute locus of Drosophila.. Cell.

[30] Leung TH, Hoffmann A, Baltimore D (2004). One nucleotide in a kappaB site can determine cofactor specificity for NF-kappaB dimers.. Cell.

[31] Han ZS, Ip YT (1999). Interaction and specificity of Rel-related proteins in regulating Drosophila immunity gene expression.. J Biol Chem.

[32] Kunsch C, Ruben SM, Rosen CA (1992). Selection of optimal kappa B/Rel DNA-binding motifs: interaction of both subunits of NF-kappa B with DNA is required for transcriptional activation.. Mol Cell Biol.

[33] Tergaonkar V, Correa RG, Ikawa M, Verma IM (2005). Distinct roles of IkappaB proteins in regulating constitutive NF-kappaB activity.. Nat Cell Biol.

[34] Jiang J, Levine M (1993). Binding affinities and cooperative interactions with bHLH activators delimit threshold responses to the dorsal gradient morphogen.. Cell.

[35] Gonzalez-Crespo S, Levine M (1993). Interactions between dorsal and helix-loop-helix proteins initiate the differentiation of the embryonic mesoderm and neuroectoderm in Drosophila.. Genes Dev.

[36] Dubnicoff T, Valentine SA, Chen G, Shi T, Lengyel JA (1997). Conversion of dorsal from an activator to a repressor by the global corepressor Groucho.. Genes Dev.

[37] Ratnaparkhi GS, Jia S, Courey AJ (2006). Uncoupling Dorsal-mediated activation from Dorsal-mediated repression in the Drosophila embryo.. Development.

[38] Valentine SA, Chen G, Shandala T, Fernandez J, Mische S (1998). Dorsal-mediated repression requires the formation of a multiprotein repression complex at the ventral silencer.. Mol Cell Biol.

[39] Heitzler P, Bourouis M, Ruel L, Carteret C, Simpson P (1996). Genes of the Enhancer of split and achaete-scute complexes are required for a regulatory loop between Notch and Delta during lateral signalling in Drosophila.. Development.

[40] Delidakis C, Preiss A, Hartley DA, Artavanis-Tsakonas S (1991). Two genetically and molecularly distinct functions involved in early neurogenesis reside within the Enhancer of split locus of Drosophila melanogaster.. Genetics.

[41] Paroush Z, Finley RL, Kidd T, Wainwright SM, Ingham PW (1994). Groucho is required for Drosophila neurogenesis, segmentation, and sex determination and interacts directly with hairy-related bHLH proteins.. Cell.

[42] Loercher A, Lee TL, Ricker JL, Howard A, Geoghegen J (2004). Nuclear factor-kappaB is an important modulator of the altered gene expression profile and malignant phenotype in squamous cell carcinoma.. Cancer Res.

