# Supplementary figures and images for: NF-κB/Rel-Mediated Regulation of the Neural Fate in Drosophila
Source: PLoS One. 2007 Nov 14;2(11):e1178. doi: 10.1371/journal.pone.0001178 (PMC2064963; doi:10.1371/journal.pone.0001178)

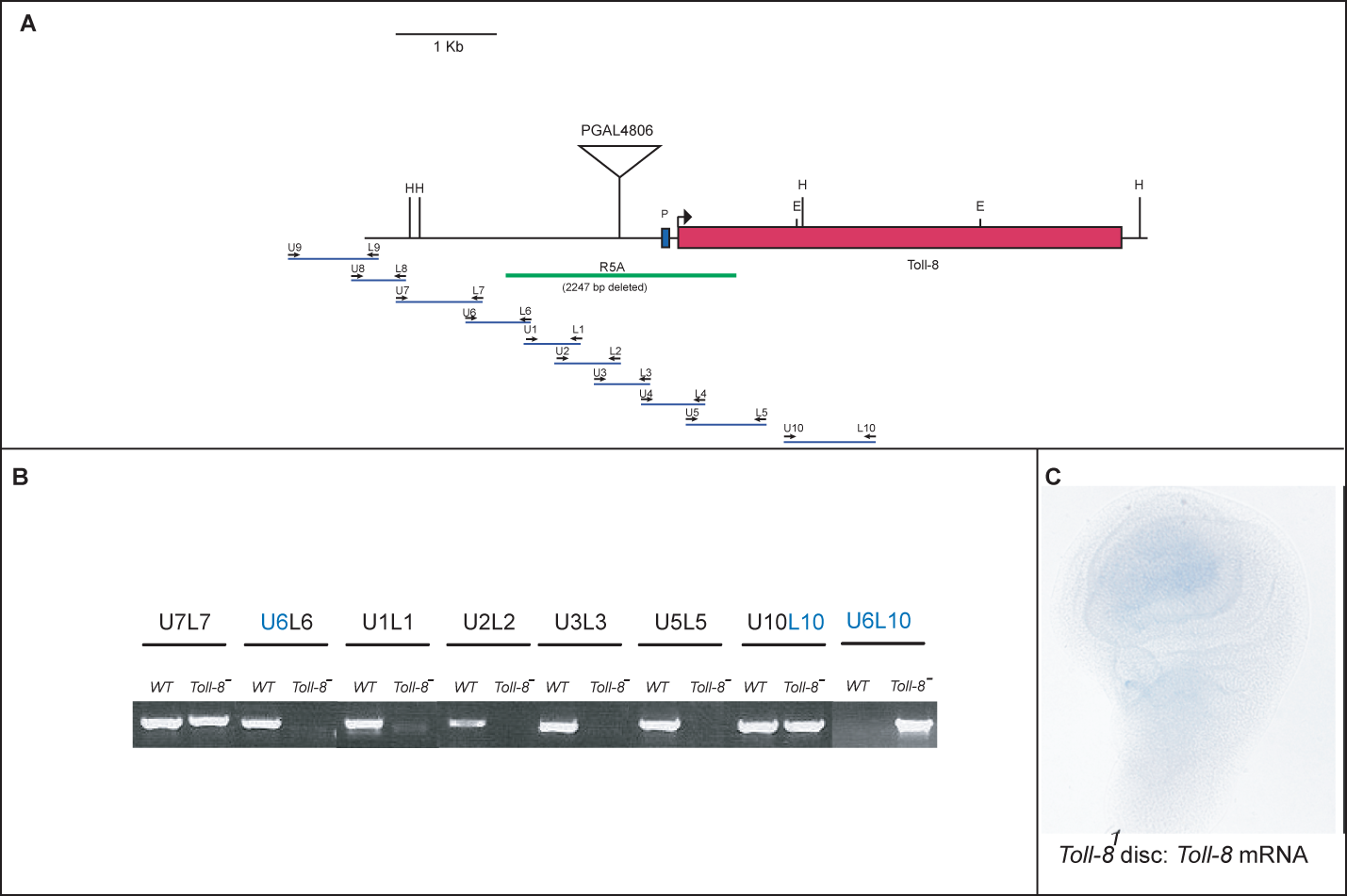

Supplement: Figure S1 — Recovery of a null allele of Toll-8. The insertion in MD806 maps 160bp upstream of the site of initiation of transcription and 640bp upstream of the translation start site and so was used to generate Toll-8 mutants by imprecise excision (A), several mutant lines were established and the extents of the deletions were confirmed by genomic PCR. Several primer pairs spanning the length of the Toll-8 gene and its upstream sequences were used to test the R5A strain and a 1.8kb U6L10 fragment normally spanning 4kb in wild-type flies was cloned and sequenced to confirm the extent of the deletion. This mutant strain was found to have a deletion of 2.24kb in the Toll-8 gene and was renamed Toll-81. (B), Toll-81 flies lack detectable transcript as judged by in situ hybridization with a U5L5 probe (C) and are predicted to be protein-null due to the absence of the usual translational start site. (0.29 MB TIF) [file pone.0001178.s001.tif]

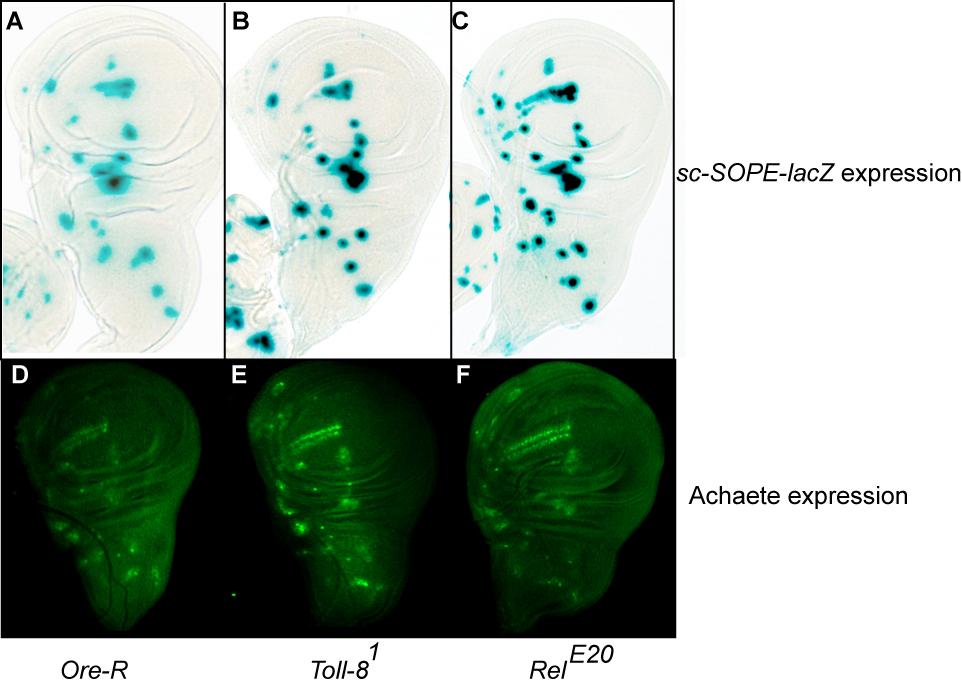

Supplement: Figure S2 — The protein products of neuronal precursor genes are confined to sensory organ precursors in Toll-8 and Relish mutants. The neuronal-specific reporter gene sc-SOPE, lacZ is found in a normal complement of precursors in wild type (A), Toll-81 (B) and RelE20 (C) mutants. sc-SOPE-lacZ contains three E-boxes, which are binding sites for bHLH proteins such as Scute and Asense. Over-expression of ase in wild-type animals leads to ectopic expression of sc-SOPE-lacZ, presumably due to generation of functional Ase protein (see Figure 3C). However, the ectopic expression of ase observed in the NF-κB mutants does not generate a corresponding global overexpression of sc-SOPE-lacZ (A–C), leading to the conclusion that Ase function in these discs is still confined to the SOPs. Staining with an antibody against Achaete shows a pattern of expression in the normal numbers and positions of the bristle precursors in wild type (D), Toll-81 (E) and RelE20 (F) mutants. (0.63 MB TIF) [file pone.0001178.s002.tif]

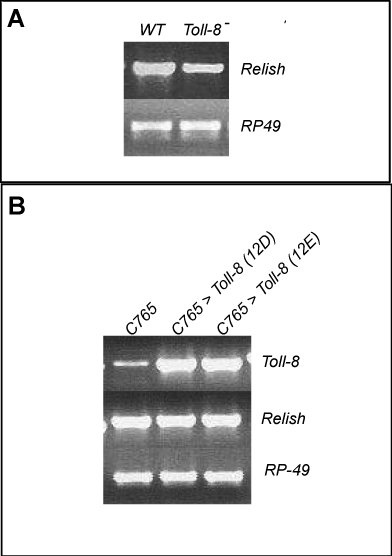

Supplement: Figure S3 — Toll-8 is required to maintain transcription of Relish.Relish transcripts are reduced in Toll-81 homozygotes (A), so Toll-8 may affect transcription of Relish. However, over-expression of Toll-8 in Gal4-C765>UAS-Toll-8 does not lead to a concomitant elevation in levels of Relish transcripts (B), indicating that the role of Toll-8 may be confined to maintenance of Relish transcript levels. (0.08 MB TIF) [file pone.0001178.s003.tif]
